# Identification of Angiogenesis-Related Prognostic Biomarkers Associated With Immune Cell Infiltration in Breast Cancer

**DOI:** 10.3389/fcell.2022.853324

**Published:** 2022-05-06

**Authors:** Dan Tao, Ying Wang, Xin Zhang, Can Wang, Dingyi Yang, Jing Chen, Yanyan Long, Yong Jiang, Xian Zhou, Ningning Zhang

**Affiliations:** ^1^ Department of Radiation Oncology, Chongqing University Cancer Hospital, Chongqing, China; ^2^ Chongqing Key Laboratory of Translational Research for Cancer Metastasis and Individualized Treatment, Chongqing University Cancer Hospital, Chongqing, China; ^3^ Department of Breast Cancer Center, Chongqing University Cancer Hospital, Chongqing, China

**Keywords:** breast cancer, angiogenesis, prognosis, risk model, immune cell infiltration

## Abstract

**Background:** This study aimed to explore the prognostic value of angiogenesis-related genes (ARGs) and their association with immune cell infiltration (ICI) in breast cancer (BC).

**Methods:** Transcriptome data of BC were obtained from the TCGA and GEO databases. Differentially expressed ARGs were identified by the limma package. The identification of key genes and construction of the risk score model were performed by univariate and multivariate Cox regression algorithms. The prognostic value of the risk score was assessed by ROC curves and nomogram. GO, KEGG pathway, and GSEA were used to investigate the biological functions of differentially expressed genes (DEGs), and CIBERSORT, ssGSEA, and xCell algorithms were performed to estimate the ICI in high-risk and low-risk groups. The correlations between prognostic biomarkers and differentially distributed immune cells were assessed. Moreover, a ceRNA regulatory network based on prognostic biomarkers was constructed and visualized by Cytoscape software.

**Results:** A total of 18 differentially expressed ARGs were identified between tumor and adjacent normal tissue samples. TNFSF12, SCG2, COL4A3, and TNNI3 were identified as key prognostic genes by univariate and multivariate Cox regression analyses. The risk score model was further constructed based on the four-gene signature and validated in GSE7390 and GSE88770 datasets. ROC curves and nomogram indicated that the risk score had good accuracy for determining BC patient survival. Biological function analysis showed that DEGs in high- and low-risk groups had a high enrichment in immune-related biological processes and signaling pathways. Moreover, significantly different ICIs were found between high- and low-risk groups, such as memory B cells, CD8^+^ T cells, resting memory CD4^+^ T cells, follicular helper T cells, regulatory T cells, monocytes, M2 macrophages, and neutrophils, and each prognostic biomarker was significantly correlated with one or more immune cell types.

**Conclusion:** The current study identified novel prognostic ARGs and developed a prognostic model for predicting survival in patients with BC. Furthermore, this study indicated that ICI may act as a bond between angiogenesis and BC. These findings enhance our understanding of angiogenesis in BC and provide novel guidance on developing therapeutic targets for BC patients.

## Introduction

The incidence of breast cancer is increasing over the world and has become the most common type of cancer in women (Siegel et al., 2021). Owing to the early diagnosis and advanced medical treatment, the 5-year relative survival rate for women with invasive breast cancer has improved from 75 to 90% over the past 25 years (Cheng, 2014), and the mortality rate of breast cancer has decreased by nearly 40% in the past 30 years (Desantis et al., 2019). However, the pace of the decline in breast cancer death rate has slowed year by year (Desantis et al., 2019), and almost all patients are at risk of treatment failure, resulting in recurrence, metastasis, and death (Riggio et al., 2021). Therefore, clarifying the interactions of key molecules during the occurrence and development of breast cancer is very essential in preventing breast cancer and finding new therapeutic targets.

Angiogenesis is a complex process of the formation of new blood vessels from preexisting vessels (Adair and Montani, 2010). When the balance between pro and antiangiogenic factors is disrupted, pathological angiogenesis develops rapidly to help cancer cells adapt cellular metabolism to cope with their high proliferation rate, making the tumor more aggressive (Viallard and Larrivee, 2017; Redfern et al., 2019). In addition, vascular networks can transport nutrients into and excrete metabolic waste from cancer cells (Hanahan and Weinberg, 2011). Tumors are incapable of growing over 1–2 mm when blood supply is deficient (Carmeliet and Jain, 2000; Li et al., 2018). In addition to vascular endothelial growth factor (VEGF)–related genes, it has been reported that other gene pathways are associated with angiogenesis and prognosis of breast cancer (Ramanathan et al., 2017; Yamada et al., 2018; Madu et al., 2020). However, a single factor may be insufficient to fully grasp the comprehensive picture of angiogenesis. Thus, greater insight may be obtained by studying multiple angiogenic factors, which would better allow dissection of these complex networks and the potential identification of hitherto unrecognized key factors for therapeutic targeting.

The mechanisms of angiogenesis in regulating tumorigenesis are very complex and not fully elucidated. Increasing evidence has shown that angiogenesis may be involved in the progression of cancer *via* interaction with the tumor immune microenvironment (TIME) (Albini et al., 2018; Chandler et al., 2019; Chen et al., 2020). It is well known that the immune surveillance system plays an important role in the clearance of abnormal cells and prevents the development of cancer (Finn, 2018). Immune checkpoint inhibitors can activate antitumor responses by blocking negative regulatory immune signals (Yi et al., 2019) and can be highly effective, particularly in the presence of significant infiltrating cytotoxic leucocytes (Yi et al., 2019). However, the response rate of immune checkpoint inhibitors in breast cancer remains lower than that in melanoma to the extent that it has only thus far found a modest role in triple-negative breast cancer (TNBC) (Longo et al., 2019). It has been reported that antiangiogenesis therapy not only prunes blood vessels which are essential to cancer growth and metastasis but also reprograms the TIME by multiple steps (Melero et al., 2014; Lanitis et al., 2015). Immune checkpoint inhibitors have been increasingly studied alongside angiogenesis in a wide variety of cancer types, including hepatocellular carcinoma (HCC), non–small cell lung cancer (NSCLC), melanoma, and breast cancer (Longo et al., 2019; Yi et al., 2019; Li et al., 2020). It is worth specifically mentioning the notable success of antiangiogenic and immunotherapy in HCC as this is now the first-line therapy and essentially constitutes one of the core rationales for this study. However, the relationship between ARGs and immune cell infiltrates (ICIs) remains unclear in breast cancer. MiRNAs are an endogenous small non-coding RNA, which play a critical role in cancer progression and are potential biomarkers and therapeutic targets. A previous study demonstrated that the competitive endogenous RNA (ceRNA) can regulate mRNA expression as “miRNA sponges”, which has crucial roles in oncogenic pathways involved in the prognosis of many types of malignant tumors (Zhang D.-D. et al., 2021). Therefore, the construction of a ceRNA network could provide new perspectives for breast cancer regulatory networks. In the present study, we aim to explore the prognostic value of ARGs in breast cancer and investigate the ICI-related mechanisms of ARGs in regulating breast cancer. Meanwhile, a ceRNA regulatory network based on the prognostic ARGs was developed. We hope our findings could provide new insights for antiangiogenic therapy and/or combination with immunotherapy for breast cancer patients.

## Materials and Methods

Data SourceRNA-Seq gene expression data of 1,049 primary breast cancer and 111 adjacent normal tissue samples were downloaded from the TCGA database (https://www.cancer.gov/about-nci/organization/ccg/research/structural-genomics/tcga). Gene expression data of 198 primary tumor samples from the GSE7390 dataset and 117 primary tumor samples from the GSE88770 dataset were downloaded from the GEO database (http://www.ncbi.nlm.nih.gov/geo). All samples were bulk tumors incorporating malignant cells and stroma. ARG sets were obtained from the Molecular Signatures Database (MSigDB) (http://www.gsea-msigdb.org/gsea/msigdb/).

### Identification of Differentially Expressed ARGs

Using the criteria of |log2 (Fold change)|> 1 and *p*-value < 0.05, differentially expressed genes (DEGs) between 1,049 bulk breast cancer tissues and 111 adjacent normal tissues in the TCGA–BRCA dataset were identified by the limma package. After overlapping with ARGs downloaded from the MSigDB database, differentially expressed ARGs were identified and used for further analysis.

### Identification of Key Prognostic ARGs in Breast Cancer

To identify differentially expressed ARGs significantly correlated with prognosis (*p* < 0.2), univariate Cox proportional hazards regression analysis was first performed. Thereafter, four key prognostic ARGs (TNFSF12, TNNI3, SCG2, and COL4A3) in breast cancer were identified by multivariate Cox regression analysis. Moreover, 1,218 samples from the TCGA database were randomly assigned to the training cohort (*n* = 975) and validation cohort (n = 243). Then, based on the four key prognostic ARGs, a logistic regression (LR) diagnostic model was constructed. To assess the performance of the LR model in both training and validation sets, the receiver operating characteristic (ROC) curves were plotted.

### Construction of the Risk Score Model and Nomogram

The risk score was calculated for each patient according to the following formula: ExpTNFSF12*Coef1 + ExpTNNI3*Coef2 + ExpSCG2*Coef3+ ExpCOL4A3*Coef4, where Exp represents the normalized expression values of each signature gene and Coef represents the regression coefficients of genes. The risk score model was initially developed on the TCGA–BRCA training set and evaluated in the GSE7390 and GSE88770 validation sets. The breast cancer patients were separated into high-risk and low-risk groups according to the median value of the risk score. Kaplan–Meier analysis (K–M) was performed to evaluate the overall survival (OS) of patients in high-risk and low-risk groups. ROC curves were plotted both in the training and validation sets to evaluate the accuracy of the risk score model. A nomogram was constructed using multivariate Cox regression analysis for clinical use. To evaluate the agreement of nomogram-predicted probability and the actual observation for OS of breast cancer patients, calibration curves were created.

### Biological Function Analysis

The clusterProfiler R package was used to analyze Gene Ontology (GO) and Kyoto Encyclopedia of Genes and Genomes (KEGG) pathway enrichment of DEGs between high- and low-risk groups. In addition, to examine the immune-related molecular mechanisms of prognostic gene signatures, gene set enrichment analysis (GSEA) was performed. The MSigDB database (http://www.gsea-msigdb.org/gsea/msigdb/) was deployed to retrieve immune-related GO gene sets.

### The Correlations Between Prognostic Signatures and Immune Cell Infiltration

First, CIBERSORT, xCell, and single-sample GSEA (ssGSEA) were carried out to quantify the immune cell infiltration in low- and high-risk groups. Specifically, the immune cell types involved in the CIBERSORT analysis were naive CD4^+^ T cells, CD8^+^ T cells, activated memory CD4^+^ T cells, resting memory CD4^+^ T cells, regulatory T cells, follicular helper T cells, gamma delta T cells, memory B cells, naive B cells, plasma cells, activated NK cells, resting NK cells, monocytes, M0 macrophages, M1 macrophages, M2 macrophages, activated dendritic cells, resting dendritic cells, activated mast cells, resting mast cells, eosinophils, and neutrophils. The immune cell types involved in the xCell analysis were B cells, memory B cells, naive B cells, plasma cells, CD8^+^ T cells, memory CD4^+^ T cells, naive CD4^+^ T cells, Tregs, Tgd cells, macrophages, M1 macrophages, M2 macrophages, NK cells, NKT cells, monocytes, DC, mast cells, eosinophils, and neutrophils. The immune cell types involved in the ssGSEA analysis were B cells, CD8^+^ T cells, aDCs, DCs, cytotoxic cells, iDCs, macrophages, mast cells, eosinophils, neutrophils, NK cells, CD56 dim NK cells, CD56 bright NK cells, pDCs, T cells, T helper cells, Tfh, Tgd, Tcm, Tem, Th1 cells, Th2 cells, Th17 cells, and Treg. Finally, Pearson’s correlations among significantly differential enriched immune cell types and prognostic ARGs were calculated.

### Construction of the ceRNA network

First, differentially expressed miRNAs and lncRNAs between the low- and the high-risk group were identified (adjusted *p*-value < 0.05). Then, the correlations among the expressions of differentially expressed miRNAs and the expressions of the four prognostic biomarkers were calculated and the negatively correlated miRNA–mRNA pairs (cor <0 and *p*-value < 0.05) were selected for further analysis. Second, the miRanda database (http://www.miranda.org) was used to predict the miRNAs targeting the four prognostic biomarkers. Then, we obtained miRNA–mRNA pairs by overlapping correlation results and miRanda predicting results. Likewise, differentially expressed lncRNAs, the expressions of which were positively correlated with the expressions of prognostic biomarkers and negatively correlated with the expressions of differentially expressed miRNAs, were used to construct the lncRNA–miRNA relationships and further overlapped with the predicted lncRNA–miRNA results by miRanda. Finally, the lncRNA–miRNA and miRNA–mRNA regulatory relationships were integrated to construct the ceRNA network by using Cytoscape.

## Results

### Identification of 18 Differentially Expressed ARGs in Breast Cancer

**FIGURE 1:**
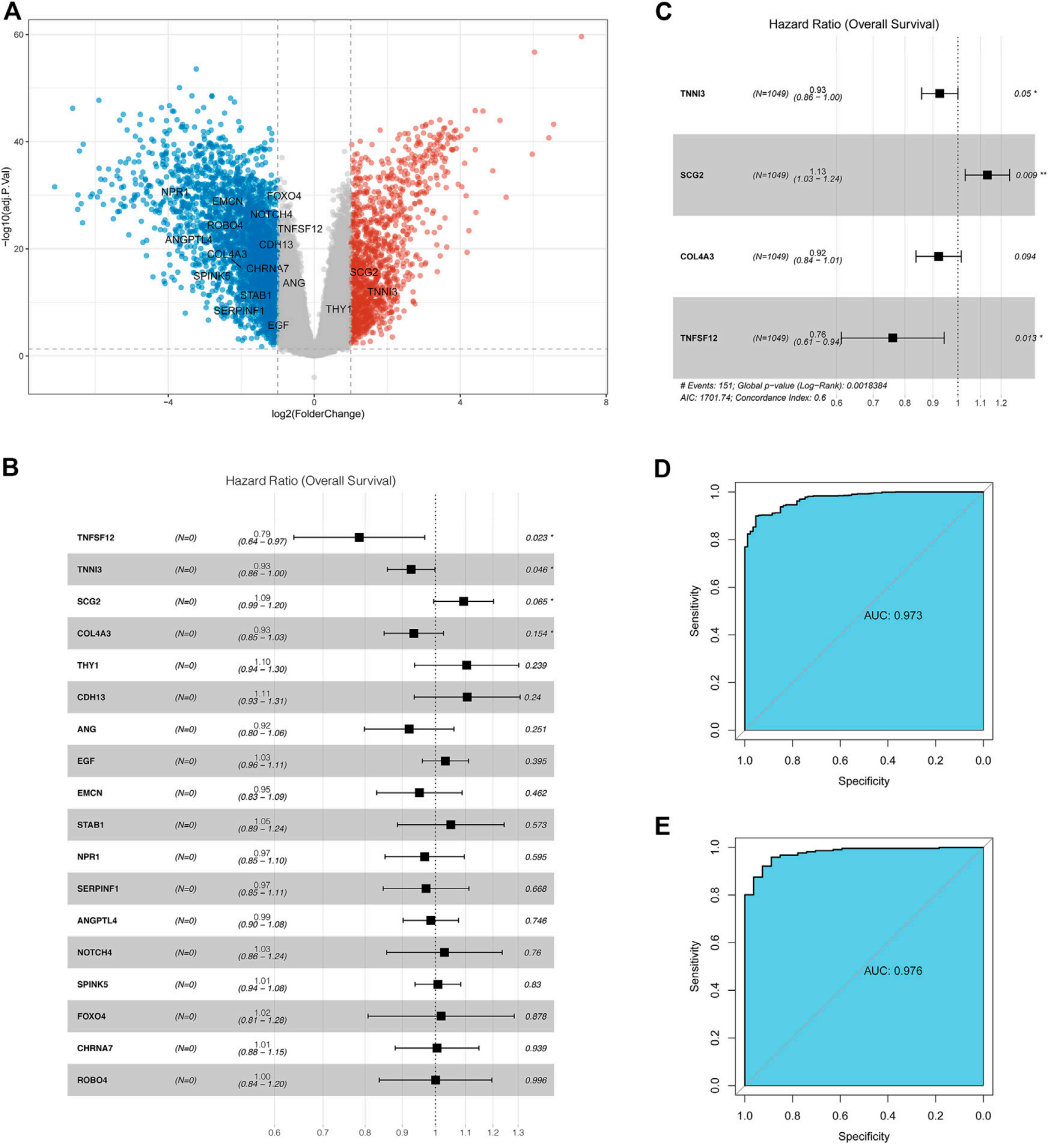
Identification of the ARGs with prognostic and diagnostic value in breast cancer patients. **(A)** Volcano plot for differentially expressed ARGs; **(B)** Univariate Cox regression analysis for differentially expressed ARGs in the TCGA database; **(C)** Multivariate Cox regression analysis for differentially expressed ARGs in TCGA datasets; **(D)** Receiver operating characteristic curve (ROC) for the diagnostic logistic regression model based on the 4-gene signatures in the training set. **(E)** ROC for the diagnostic logistic regression model based on the 4-gene signatures in the validation set.

A total of 4,003 DEGs including 1,241 upregulated and 2,762 downregulated genes were identified between tumor and adjacent normal tissue samples (Supplementary Table S1). Eighteen differentially expressed ARGs were identified, including three upregulated and 15 downregulated ARGs in tumor samples relative to adjacent normal tissue samples (Figure 1A).

### Identification of Key Prognostic ARGs in Breast Cancer

Thereafter, the prognostic value of 18 ARGs identified in BC was explored by univariate Cox regression analysis. In this stage analysis, clinicopathological factors (such as grade, lymph node status, and tumor size, etc.) were not included. The results showed that TNFSF12, TNNI3, SCG2, and COL4A3 were significantly associated with prognosis (*p* < 0.2), among which TNFSF12, TNNI3, and COL4A3 played a protective role (HR < 1) and SCG2 acted as a risk factor (HR > 1, Figure 1B). Multivariate Cox regression analysis was performed using these four genes to obtain more robust signature genes. TNFSF12, TNNI3, SCG2, and COL4A3 were still strongly correlated with prognosis and identified as key prognostic genes (Figure 1C). The coefficients of each gene are displayed in Supplementary Table S2. Furthermore, we constructed a diagnostic LR model based on the four gene signatures and found that the LR model had a good performance in classifying breast cancer patients both in the training set (area under the ROC curve = 0.973, Figure 1D) and in the validation set (area under the ROC curve = 0.976, Figure 1E).

### Construction and Validation of the ARG-Based Prognostic Risk Score Model

**FIGURE 2:**
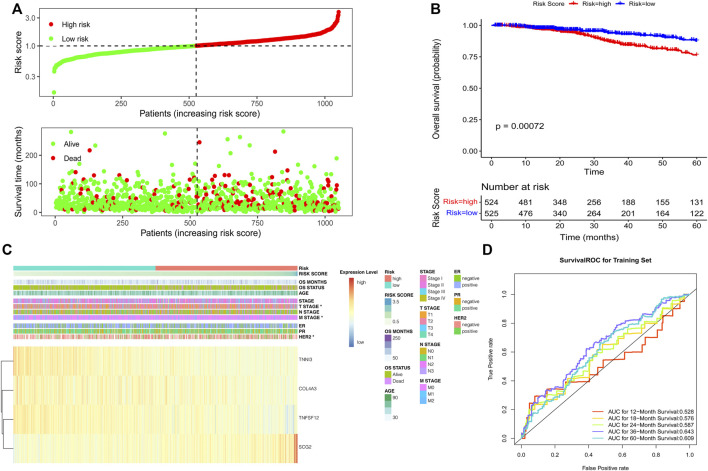
Development of the risk score model based on the four ARG signature in patients with breast cancer from TCGA datasets. **(A)** Risk score distribution and survival status of patients; **(B)** Kaplan–Meier analysis of the prognostic model; **(C)** Heatmap of the four ARG expression and clinical parameter profiles between high- and low-risk groups; **(D)** Time-dependent ROC analysis of the risk score model.

The risk score of individual patients was calculated based on the coefficients of TNFSF12, TNNI3, SCG2, and COL4A3 in Supplementary Table S2. According to the median of the risk scores of the patients in the TCGA training set, high- and low-risk groups were divided (Figure 2A). A significant difference in the 5-year overall survival was observed between these two groups (*p* = 0.00072) (Figure 2B). The expressions of TNFSF12, TNNI3, SCG2, and COL4A3 and clinical characteristics in high- and low-risk groups are displayed in the heatmap (Figure 2C). The ROC curves revealed that the risk score model proved to be significantly powerful in predicting the survival of BC patients. The areas under the ROC curves (AUC) were 0.643 for 3-years and 0.609 for 5-years overall survival (Figure 2D). The consensus results were also obtained in the GSE7390 (Supplementary Figures S1A–D) and GSE88770 (Supplementary Figures S2A–D) validation sets.

**FIGURE 3:**
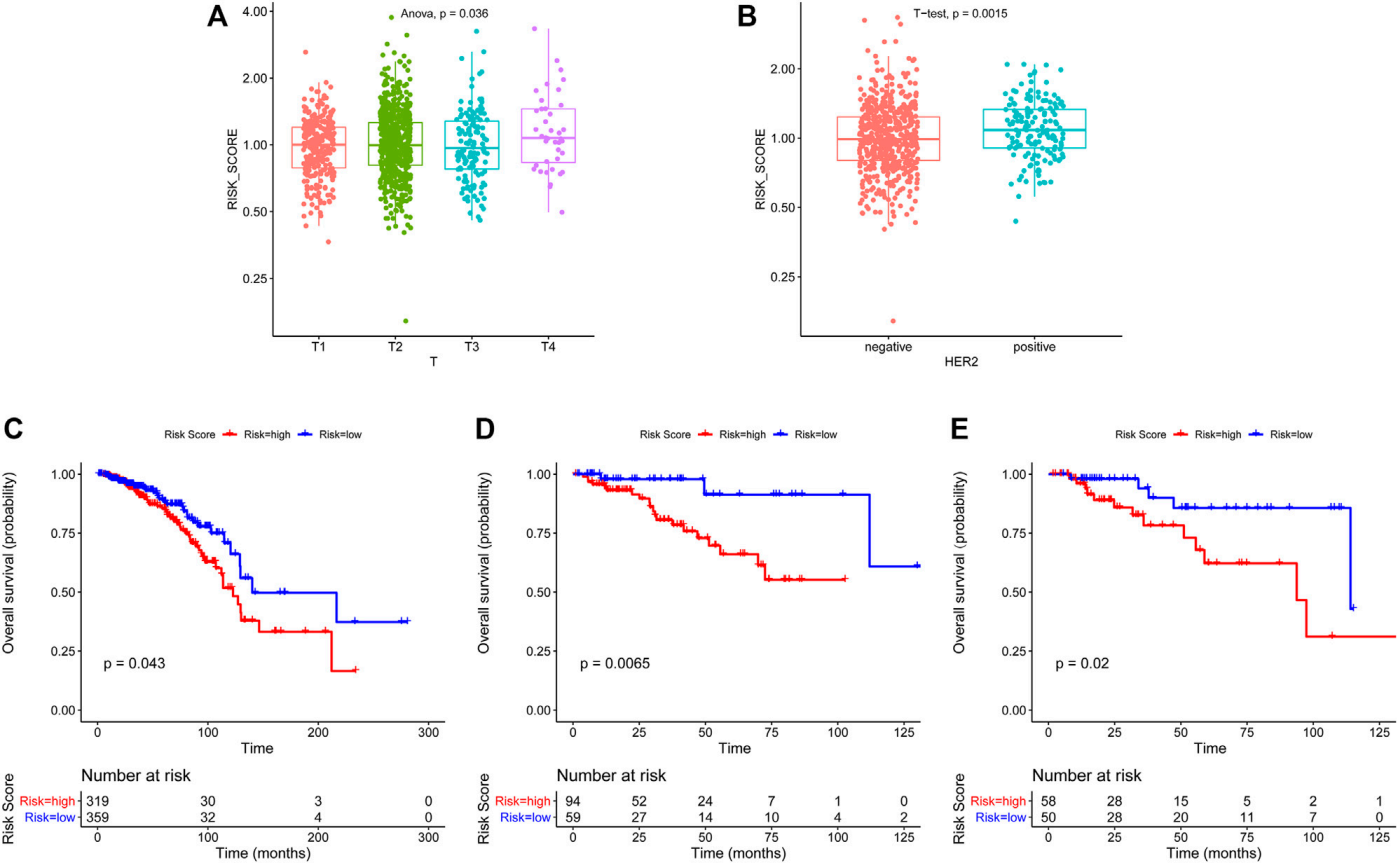
Association of clinicopathological features with ARG-based risk score and overall survival analysis in different breast cancer subtypes based on ARG-based risk score. The ARG-based risk score was associated with T stage **(A)** and HER2 status **(B)**. Kaplan–Meier curves of overall survival for HR + HER2- **(C)**, HER2+ **(D)**, and TNBC **(E)** subtypes based on ARG-based risk score. *p*-values were calculated using the log-rank test.

Then, we investigated the association between risk score and clinical features, including age, staging, estrogen receptor (ER), progesterone receptor (PR), and human epidermal growth factor receptor 2 (HER2) status. We found that breast cancer patients with advanced T stage and HER2-positive status had higher risk scores (Figures 3A, B), while no significant difference in risk scores was observed in other groups (Supplementary Figures S3A–F). Moreover, we further investigate whether this ARG-based scoring model would work in different molecular subtypes, including hormone receptor (HR) positive/HER2 negative, HER2 positive, and TNBC. The results showed that in all subtypes, patients with low risk had significantly longer survival (Figures 3C–E).

### Construction and Analysis of the Nomogram

**FIGURE 4:**
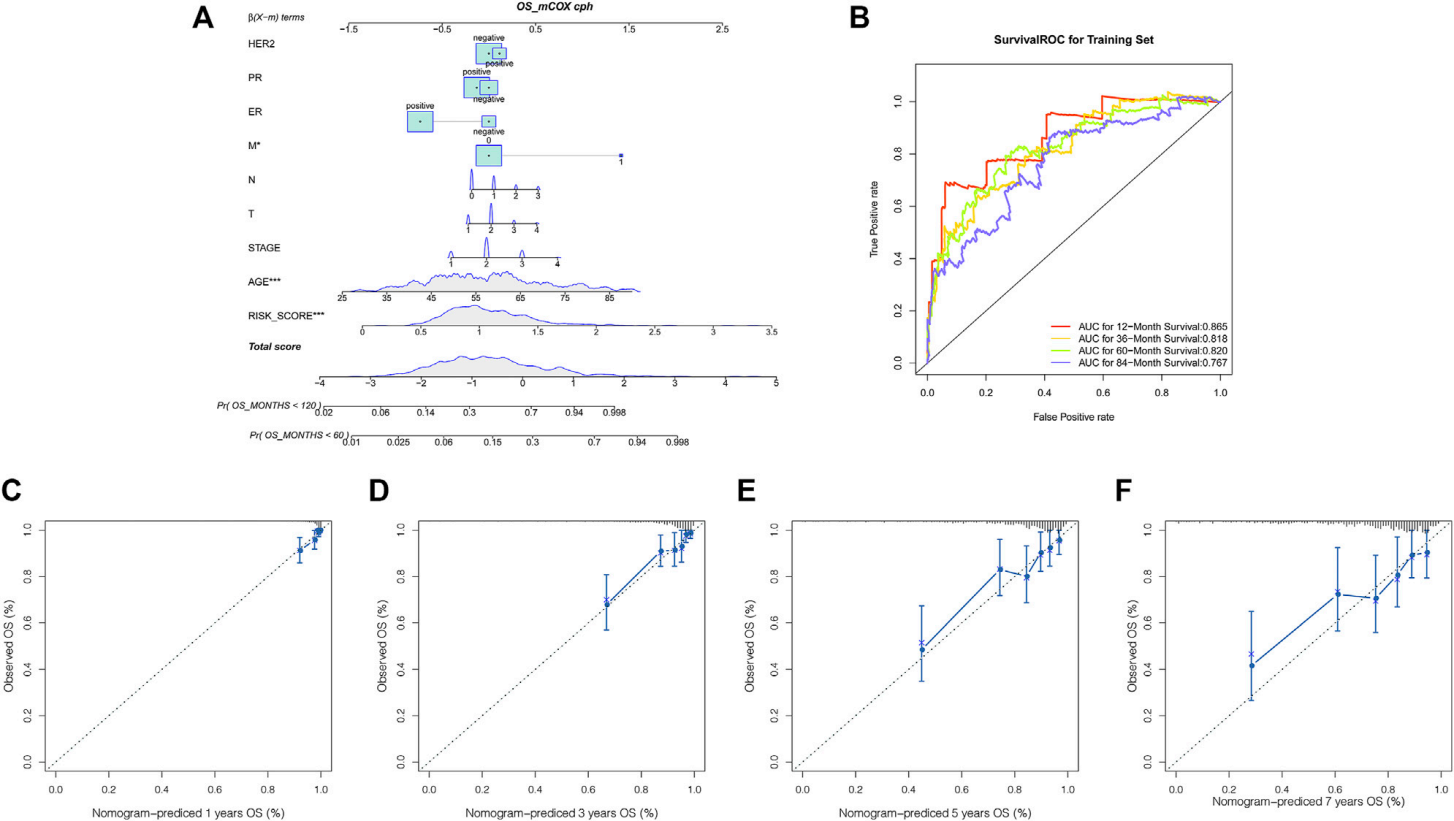
Nomogram for predicting the overall survival probability of breast cancer patients. **(A)** Nomogram was built to quantify survival probability for individual breast cancer patients based on the risk score and clinical variables. Survival time is measured in months; **(B)** ROC analysis of the nomogram for predicting the 1-, 3-, 5- and 7- year OS; **(C–F)** calibration curves of the nomogram for predicting the 1-year, 3-years, 5- year, and 7-year survival probability.

Furthermore, we performed multivariate analysis using the abovementioned clinical characteristics and risk score as factors to construct a nomogram (Figure 4A). ROC analysis was performed to evaluate the prognostic value of our model. The area under the curve (AUC) for our model was 0.865 at 1 year, 0.818 at 3 years, 0.820 at 5 years, and 0.767 at 7 years (Figure 4B). The calibration curves for the probability of 1-, 3-, 5-, and 7-year OS revealed good concordance between nomogram prediction and actual observations (Figures 4C–F), indicating the clinical use of the nomogram.

### Biological Function Analysis of the Four Prognostic ARGs and DEGs in the Low- and High-Risk Groups

**FIGURE 5:**
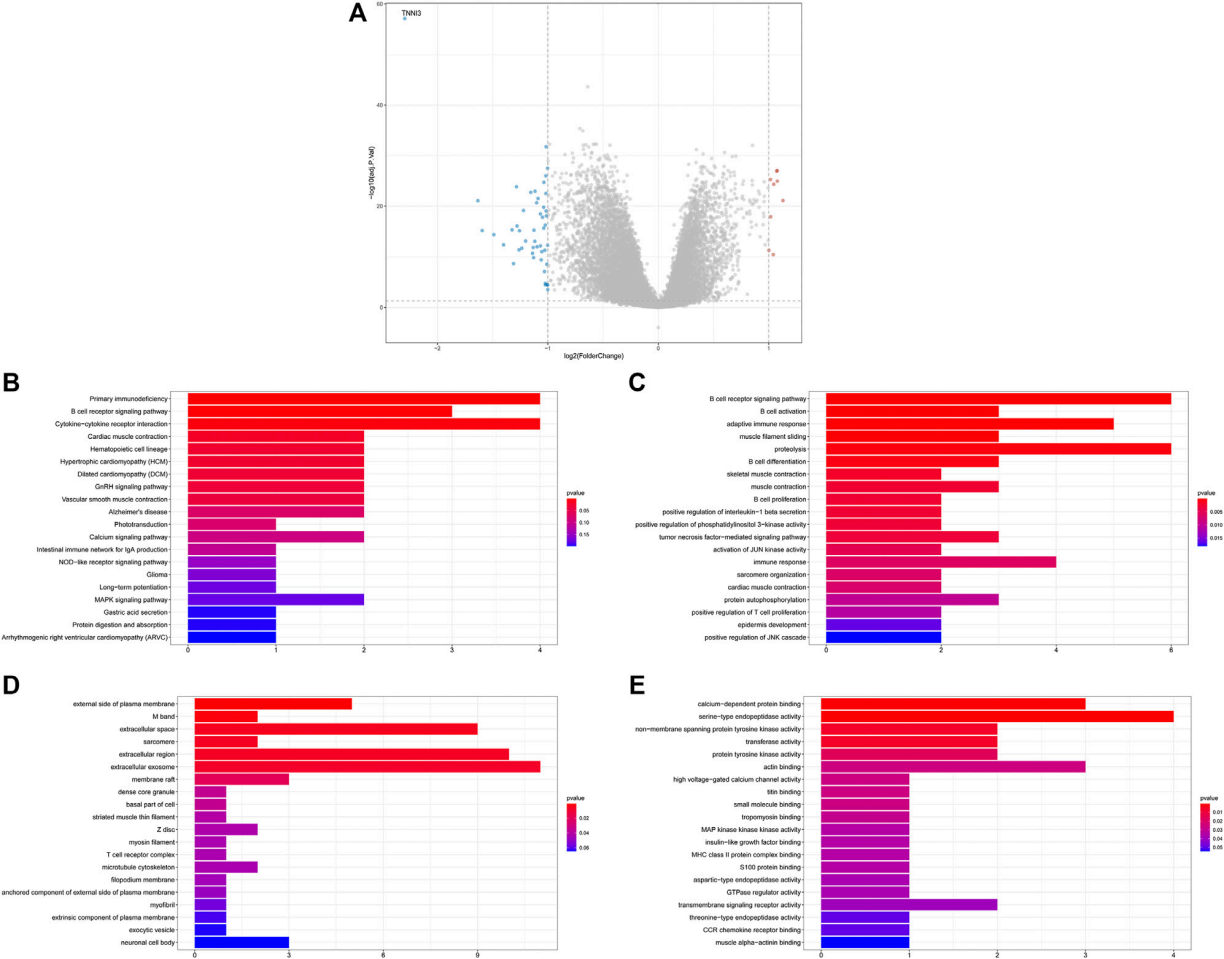
Functional annotation of differentially expressed genes (DEGs) between the low- and high-risk groups. **(A)** Volcano plot for DEGs between low- and high-risk groups; **(B)** top 20 KEGG pathways; **(C–E)** top 20 GO terms, including biological processes **(C)**, cellular components **(D)**, and molecular functions **(E)**.

**FIGURE 6:**
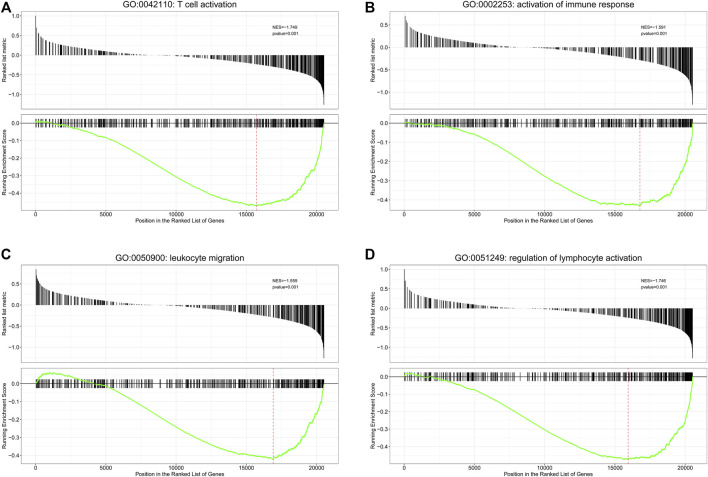
Gene set enrichment analysis (GSEA) analysis for biological processes enriched in low- and high-risk groups. GSEA plots with normalized enrichment score (NES) and *p*-value are shown here for immune-related genes sets, where significant enrichment was observed. Negative NES refers to enrichment in the low-risk group. **(A)** T cell activation; **(B)** activation of immune response; **(C)** leukocyte migration; **(D)** regulation of lymphocyte activation.

First, we performed function enrichment of these four prognostic ARGs. The results indicated that COL4A3, SCG2, and TNFSF12 were involved in the pathway of regulation of endothelial cell proliferation and regulation of epithelial cell proliferation. TNNI3, SCG2, and TNFSF12 were involved in the pathway of blood vessel morphogenesis, blood vessel development, vasculature development, and tube morphogenesis (Supplementary Figure S4; Supplementary Table S3). Furthermore, 57 DEGs including nine upregulated and 48 downregulated genes were identified between the low- and high-risk groups (Figure 5A). To explore the molecular mechanisms underlined, GO analysis and KEGG pathway enrichment analysis of DEGs were performed. The top 20 GO terms and top 20 KEGG pathways (Figure 5B), including biological processes (Figure 5C), cellular components (Figure 5D), and molecular functions (Figure 5E), are displayed in the bar plot. Moreover, GSEA analysis revealed that DEGs were involved in many immune-related biological processes (Supplementary Table S4), including T cell activation (Figure 6A), activation of immune response (Figure 6B), leukocyte migration (Figure 6C), and regulation of lymphocyte activation (Figure 6D).

### The Correlations Among Immune Cell Infiltration and Prognostic Biomarkers

**FIGURE 7:**
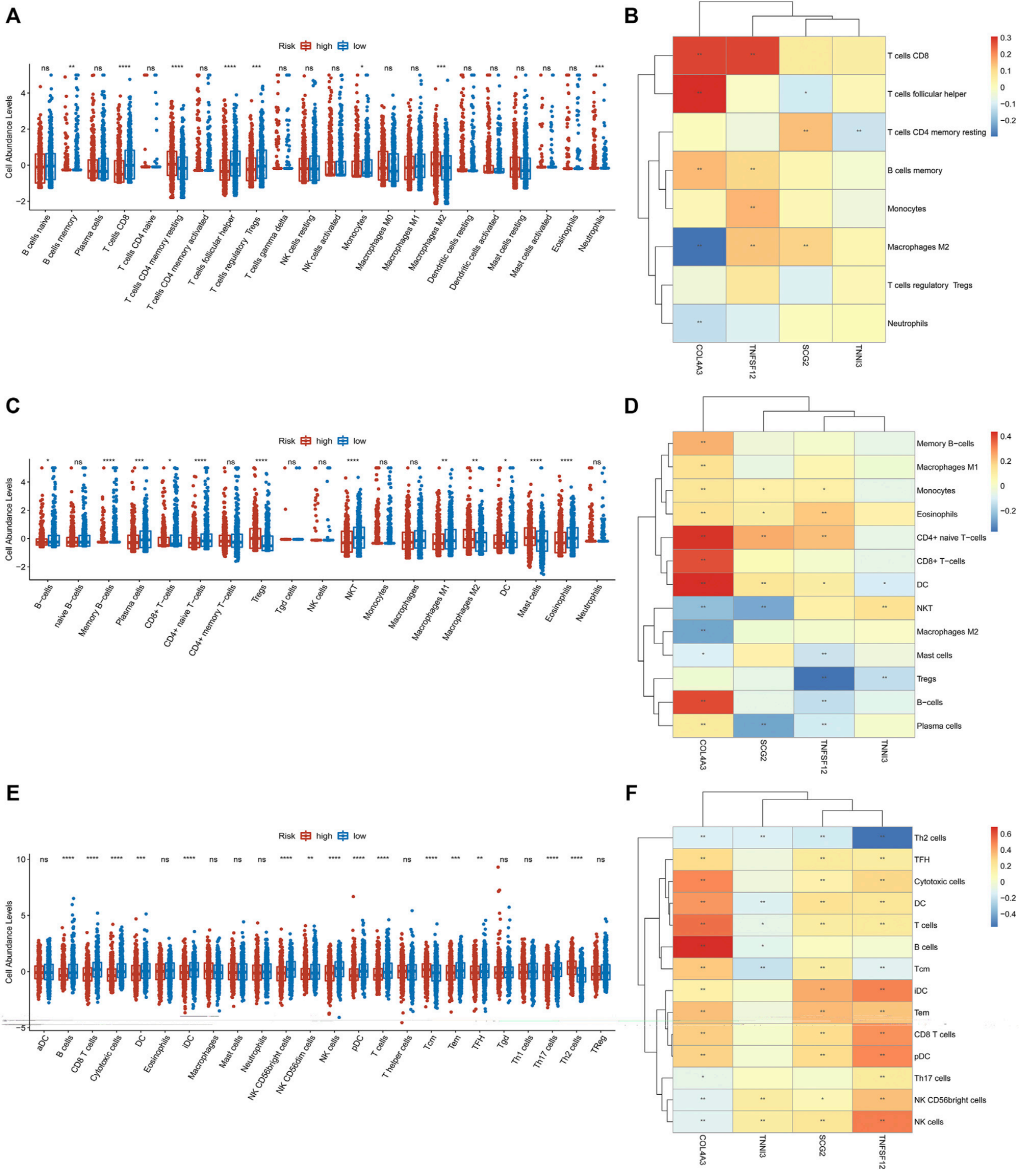
CIBERSORT, ssGSEA, and xCell were used to estimate the immune cell infiltration in high- and low-risk groups, respectively **(A,C,E)**. The correlations among immune cell infiltration and the four prognostic ARGs are displayed in the heatmaps **(B,D,F)**.

Previous studies revealed that immune cells in the tumor microenvironment (TME) can regulate angiogenesis (Albini et al., 2018). Therefore, we explored the correlations among immune cells and the four prognostic biomarkers in breast cancer. To obtain more comprehensive results, we used different methods to evaluate immune cell infiltration in low- and high-risk groups. As for CIBERSORT, memory B cells, CD8^+^ T cells, follicular helper T cells, regulatory T (Tregs) cells, and monocytes were significantly higher in low-risk groups, while the infiltration of resting memory CD4^+^ T cells, M2 macrophages, and neutrophils was markedly elevated in high-risk groups (Figure 7A). TNFSF12 was significantly positively correlated with memory B cells, monocytes, M2 macrophages, and CD8^+^ T cells. TNNI3 was significantly negatively correlated with resting memory CD4^+^ T cells. SCG2 was significantly positively associated with resting memory CD4^+^ T cells and M2 macrophages and significantly negatively associated with follicular helper T cells. COL4A3 was significantly positively correlated with follicular helper T cells, CD8^+^ T cells, and memory B cells and significantly negatively correlated with neutrophils and M2 macrophages (Figure 7B). As for xCell and ssGSEA analysis, we found that 12 and 15 immune cell types were significantly differentially distributed between low- and high-risk groups (Figures 7C,E), respectively. Meanwhile, the relationship between the prognostic biomarkers and differentially distributed immune cells is displayed in the heatmaps (Figures 7D,F).

### Construction of ceRNA Network Based on Prognostic Biomarkers

**FIGURE 8:**
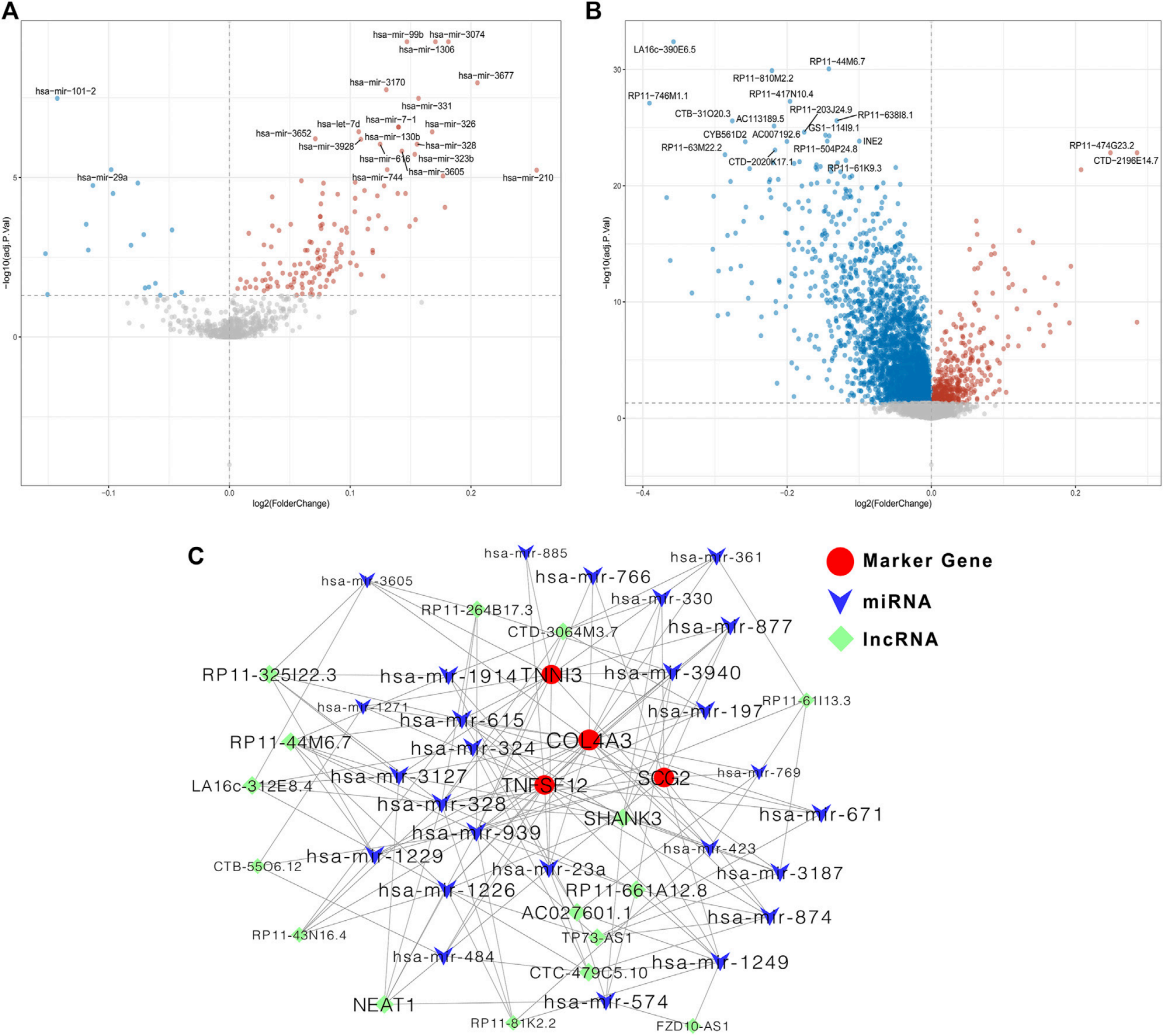
Construction of the ceRNA regulatory network based on the four prognostic biomarkers. **(A)** Volcano plot for differentially expressed miRNAs between low- and high-risk groups; **(B)** volcano plot for differentially expressed lncRNAs between low- and high-risk groups; **(C)** ceRNA regulatory network among miRNAs, lncRNAs, and mRNA of the four prognostic ARGs.

Last, we constructed a ceRNA regulatory network based on the four prognostic biomarkers. 158 differentially expressed miRNAs were identified between the low- and high-risk groups (Figure 8A). Then, the correlations between the expressions of differentially expressed miRNAs and the expressions of the four prognostic biomarkers were calculated (Supplementary Table S5), and a total of 294 negatively correlated miRNA–mRNA pairs were obtained. After overlapping with the predicting miRNA–mRNA pairs by miRanda, a total of 111 miRNA–mRNA pairs were identified for further use. Meanwhile, 3,718 differentially expressed lncRNAs were identified between the low- and high-risk groups (Figure 8B). The expressions of 3,454 lncRNAs were positively correlated with at least one prognostic biomarker (Supplementary Table S6), and 713 negatively correlated lncRNA–miRNA pairs were obtained (Supplementary Table S7). After overlapping with the predicting lncRNA–miRNA pairs by miRanda, a total of 518 lncRNA–miRNA pairs were identified for further use. Then, we constructed and visualized the ceRNA network by using Cytoscape software by filtering out the degrees of nodes <5 (Figure 8C).

## Discussion

Angiogenesis plays an essential role in promoting tumor growth and metastasis (Madu et al., 2020). Tumor angiogenesis involves not only cancer cells but also immune infiltrating cells, which are the important components of the TME (Albini et al., 2018; Larionova et al., 2021). There is accumulating evidence that angiogenesis and immune cells are interconnected and facilitated by shared regulators in cancer (Motz and Coukos, 2011). In this study, a breast cancer risk model based on ARGs was developed and validated. We found that genes in high- and low-risk groups were significantly enriched into immune-related biological processes and signaling pathways. Moreover, between high- and low-risk groups, significantly different immune cell infiltration was observed and strongly associated with prognostic ARGs.

TNFSF12, TNNI3, SCG2, and COL4A3 were identified as prognostic biomarkers in breast cancer by univariate and multivariate Cox regression algorithms. Meanwhile, functional enrichment analysis revealed that these four genes were involved in multiple pathways of angiogenesis. TNFSF12, also known as TWEAK or CD255, belongs to the tumor necrosis factor (TNF) superfamily, which is expressed in various types of cancer and has been reported to stimulate tumor growth and angiogenesis (Ho et al., 2004; Kawakita et al., 2004; Shimada et al., 2012). Meanwhile, previous studies also indicated that TNFSF12 might play a pro-tumorigenic role in human breast cancer (Michaelson et al., 2005). However, TNFSF12 could also induce multiple pathways of cell death, including caspase-dependent apoptosis, cathepsin B–dependent necrosis, and endogenous TNF-alpha–mediated cell death, in a cell type–specific manner (Nakayama et al., 2003; Ikner and Ashkenazi, 2011), and studies have shown that TNFSF12 could promote cell death in human peripheral blood mononuclear cells (Kaplan et al., 2002), human colonic adenocarcinoma cells (Kawakita et al., 2005), and human breast adenocarcinoma cells (Marsters et al., 1998). In the current study, we found that TNFSF12 acts as a protective factor in breast cancer and that poor survival of breast cancer patients was related to decreased expression of TNFSF12. All these indicate that further studies are needed to elucidate the specific role and mechanism of TNFSF12 in breast cancer.

TNNI3 belongs to the sarcomere gene and is well-acknowledged to play a critical role in the development of ventricular hypertrophy and is a causative factor for hypertrophic cardiomyopathy (Lopes et al., 2013; Phan et al., 2014). TNNI3 overexpression was commonly observed in various solid tumors and involved in the progression and metastasis of ovarian cancer (Salvesen and Trovik, 2011; Chen et al., 2014; Yang et al., 2017; Yin et al., 2021). Previous studies indicated that TNNI3 was predicted as a target of hsamiR-375 in various stages of laryngeal squamous cell carcinoma (LSCC) (Yu et al., 2018). Some studies suggested the potential use of TNNI3 as a marker or targeted therapy for cancer. But on the other hand, in patients with TNNI3 elevation, careful attention must be paid to the cardiotoxicity of anticancer therapy (Chen et al., 2014). In this study, we first reported that TNNI3 was downregulated in breast cancer and was a beneficial prognostic marker. However, the role in tumor angiogenesis and the function in breast cancer of TNNI3 is still unknown, which requires further investigations.

SCG2, a member of the chromogranin/secretogranin family of neuroendocrine secretory proteins, is essential for endothelial angiogenesis and new blood vessel formation (Luo et al., 2020). SCG2 had been reportedly upregulated in many tumors, including olfactory neuroblastomas (Topcagic et al., 2018), pancreatic cancer (Alrawashdeh et al., 2019), prostatic small-cell neuroendocrine carcinoma (Clegg et al., 2003), small intestinal neuroendocrine neoplasia, kidney renal clear cell carcinoma (KIRC) (Yang et al., 2020), NSCLC (Cury et al., 2019; Wang and Chen, 2020), and colorectal cancer (CRC) (Sun et al., 2019; Liu et al., 2020), and the increased expression of SCG2 was correlated with good survival outcomes for KIRC patients (Yang et al., 2020) but associated with decreased survival in Group 3 medulloblastoma (Thompson et al., 2017) and NSCLC (Cury et al., 2019; Wang and Chen, 2020) and CRC patients (Sun et al., 2019; Liu et al., 2020). However, Fang et al. recently reported that in malignant CRC tissues, SCG2 was significantly downregulated and patients with higher expression of SCG2 had longer disease-free survival and OS. In addition, higher expression of SCG2 impaired tumor growth and angiogenesis by promoting the degradation of hypoxia-inducible factor-1α in CRC (Fang et al., 2021). For the first time, the current study reported that SCG2 had a higher expression level and acted as an unfavorable prognostic marker in breast cancer. However, the detailed function of SCG2 in breast cancer has not been well-defined.

COL4A3, an adhesion molecule, is involved in basement membrane development (Siamakpour-Reihani et al., 2015). A previous study demonstrated that the α3 (IV) chain encoded by COL4A3 could produce tumstatin, which can impede blood vessel formation *in vivo* and prevent tumor proliferation and metastasis (Hamano et al., 2003), and a significant association was observed between the abnormal expression of COL4A3 and tumor size, tumor grade, metastasis, invasion, and prognosis in several malignancies (Nie et al., 2013; Yang et al., 2021). However, the role of COL4A3 in prognosis is inconsistent in different tumors. For example, COL4A3 was downregulated and positively correlated with better prognosis in lung, colon, bladder, salivary gland, and nasopharyngeal carcinoma cancers (Orth et al., 1977; Alampi et al., 1989; Karja et al., 1995; Metodieva et al., 2011; Deng et al., 2014; Liang et al., 2020), while other studies reported that patients with higher expression of COL4A3 had a significantly worse OS in high-grade serous ovarian cancer (HGSC) (Siamakpour-Reihani et al., 2015) and gastric carcinoma (Nie et al., 2013). In the current study, a significant positive association was found between higher COL4A3 expression and a favorable prognosis in breast cancer, but the exact mechanisms need further study.

There is one noteworthy point that should not be overlooked in the current study. Based on the four key prognostic ARGs, a logistic regression diagnostic model was constructed, and the results indicated that the AUC of the diagnostic model was higher than that of the risk score model. This result seems confusing, but it is reasonable. It is well known that the purposes of diagnostic and prognostic models are different. The main purpose of the diagnostic model is for classification, while prognostic models incorporate the dimension of time, adding a stochastic element. The ROC curve is typically used to evaluate clinical utility for both diagnostic and prognostic models. The ROC curve is very useful for classification in the diagnostic model. However, the ROC curve is not the only evaluation metric for prognostic models. The evaluation of prognostic models should combine the ROC curve with discrimination and calibration (Cook, 2008), while our prognostic models demonstrated good calibration and discriminatory abilities. In summary, the AUC of diagnostic and prognostic models should be interpreted separately rather than comparably. Moreover, in the current study, the ARG-based risk scores were associated with HER2 status, which is consistent with the fact that HER2 breast cancer is associated with a more aggressive pattern. However, the survival of patients with high risks was worse, no matter what kind of molecular subtype. All these results indicated that our angiogenesis risk model had universal applicability for breast cancer and suggested that the angiogenesis-related prognostic genes might be involved in the occurrence and development of breast cancer, but the specific molecular mechanism needs further experiments *in vivo* and *in vitro*.

To understand the potential molecular mechanism of ARGs involved in breast cancer, we performed KEGG and GO analyses and found that DEGs between low- and high-risk groups were significantly enriched in many immune-related pathways, including primary immunodeficiency, B cell receptor signaling pathway, and adaptive immune response. Furthermore, GSEA also revealed the involvement of immune-related signaling in the low-risk group, such as T cell activation, activation of the immune response, leukocyte migration, and regulation of lymphocyte activation. These findings implied that prognostic ARGs have a close relationship with immune function in breast cancer. Thus, we further analyzed the difference in immune cell infiltration between high- and low-risk groups using CIBERSORT, xCell, and ssGSEA analysis. All these analyses indicated that immunoreactive cells (such as CD8^+^ T cells, NKT cells, and DC cells) were decreased and immunosuppressive cells (such as macrophages M2) were increased in the high-risk group, which resulted in a state of immunosuppression and might lead to adverse prognosis of these patients. In addition, we also found that the expression of these four ARGs individually was closely related to the infiltration of immune cells. All of these results further demonstrated the interaction between ARGs and immune infiltrates in breast cancer. Notably, in the current study, we noticed that patients in the high-risk group had reduced T cell infiltration, and T cell activation was significantly enriched in the low-risk group. To date, in both preclinical and clinical studies, T cell activation, in particular, has been used to evaluate the efficacy of immune checkpoint blockade (Zheng et al., 2018). Moreover, there are signs that the tumor vasculature itself constitutes a substantial barrier to T cells (Lanitis et al., 2015). However, whether a different immune population impacts ARGs or vice versa is unclear, and whether a particular immune cell type is more involved in the breast cancer tumorigenic process and affects the therapeutic effect of antiangiogenesis and/or immunotherapy is unknown. Thus, further research on the mechanism is warranted to clarify these questions.

Given the essential factors required for angiogenesis in tumor development, growth, and metastatic spread, antiangiogenic therapy has been widely studied for a long time. However, antiangiogenic treatment is not currently the standard of care in breast cancer as the lead agent, bevacizumab, did not show survival advantage (Zhang M. et al., 2021). Antiangiogenic agents can enhance effector immune cell infiltration by inducing vascular normalization and reducing immunosuppression (Chen et al., 2021). Emerging evidence has shown that antiangiogenic agents could enhance the effect of immunotherapy with the continuous development of immunotherapy drugs and antiangiogenic agents (Esteva et al., 2019; Anderson et al., 2022). Currently, in breast cancer, especially in TNBC, multiple clinical trials of the application of combined immune checkpoint inhibitors with antiangiogenic drugs, such as multitarget receptor tyrosine kinase inhibitor anlotinib and small-molecule tyrosine kinase inhibitor apatinib (VEGFR2 inhibitor), which are manufactured in China, are being carried out (NCT03855358, NCT04914390, NCT04877821, NCT04405505, NCT04722718, NCT04303741, NCT03945604, and NCT03394287, et al.). Under such circumstances, how antiangiogenic treatment interacts with the ARGs and immune cell infiltration in terms of benefit would be interesting and meaningful to provide new insights into tumor angiogenesis and their clinical implications.

The advantages of this study are that TCGA data sets with pairs of breast cancer and adjacent normal tissues were used, which avoids cancer heterogeneity and ensures reliable DEG results. Meanwhile, we identified significant DEGs related to breast cancer survival and then constructed a gene signature with prognostic value. Moreover, to prove the robustness of our four-gene signature, two independent GEO cohorts were used as validation data sets. However, our study has several limitations worth noting. First, the treatment schemes of the breast cancer patients were unknown, that is, adjuvant chemotherapy, trastuzumab of targeted therapy, or hormone therapy, which are associated with breast cancer outcomes. Second, we did not validate prognostic gene expression at the protein level in breast tumor specimens. This is the main limitation of this study. Further studies to explore the expression and functions of these four genes in breast cancer are warranted. Third, immune population assessments are surrogates from the transcriptome data of the database. Confirming the most notable differences in immune cell subtypes by direct pathological visualization would be good. Direct immune cell population assessments are required in further studies. Fourth, the direction of effect is unknown, that is, whether the ARGs affect the composition of immune cell infiltrates or whether immune infiltrates influence the levels of or even directly express ARGs. Further functional manipulation experiments are required to elucidate the nature of these interrelationships.

Taken together, the current study aimed to explore the prognostic value of ARGs and their connections with immune cell infiltration in patients with breast cancer. We identified four prognostic ARG biomarkers in breast cancer and established an accurate risk model and nomogram for predicting survival in patients with breast cancer. Moreover, we also found that immune cell infiltration may act as a bond between angiogenesis and breast cancer. Further *in vivo* and *in vitro* experiments will be carried out to unveil the molecular mechanisms of those ARGs in regulating breast cancer. These findings may enhance our understanding of angiogenesis in breast cancer and provide extensive and new insights on breast cancer therapy.

## Data Availability

The datasets presented in this study can be found in online repositories. The names of the repository/repositories and accession number(s) can be found in the article/Supplementary Material.

## Supplementary Material

Supplementary Figure S1 Development of a risk score model based on the four ARG signature in patients with breast cancer from GSE7390 datasets. **(A)** Risk score distribution and survival status of patients; **(B)** Kaplan–Meier analysis of the prognostic model; **(C)** Heatmap of the four ARG expression and clinical parameter profiles between high- and low-risk groups; **(D)** Time-dependent ROC analysis of the risk score model.

Supplementary Figure S2 Development of a risk score model based on the four ARG signature in patients with breast cancer from GSE88770 datasets. **(A)** Risk score distribution and survival status of patients; **(B)** Kaplan–Meier analysis of the prognostic model; **(C)** Heatmap of the four ARG expression and clinical parameter profiles between high- and low-risk groups; **(D)** Time-dependent ROC analysis of the risk score model.

Supplementary Figure S3 Relationship between ARG-based risk score and clinical features including M stage **(A)**, age **(B)**, N stage **(C)**, clinical stage **(D)**, ER status **(E)**, and PR status **(F)**.

Supplementary Figure S4 Function enrichment of these four prognostic genes (TNFSF12, SCG2, COL4A3, and TNNI3).
