# Supplementary figures and images for: Identification of Angiogenesis-Related Prognostic Biomarkers Associated With Immune Cell Infiltration in Breast Cancer
Source: Front Cell Dev Biol. 2022 May 6;10:853324. doi: 10.3389/fcell.2022.853324 (PMC9121305; doi:10.3389/fcell.2022.853324)

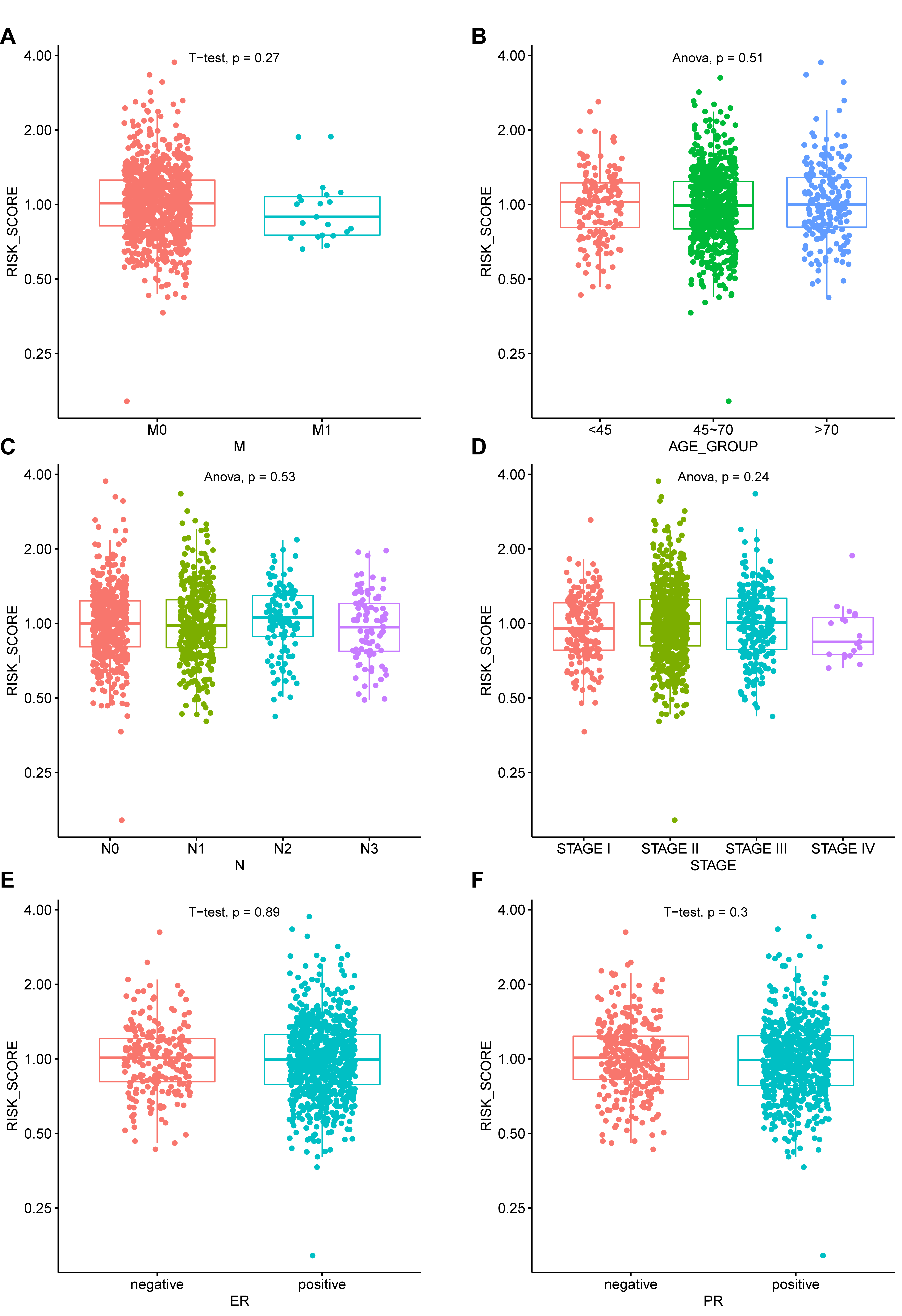

Supplement: Supplementary file 5 [file Image3.TIF]

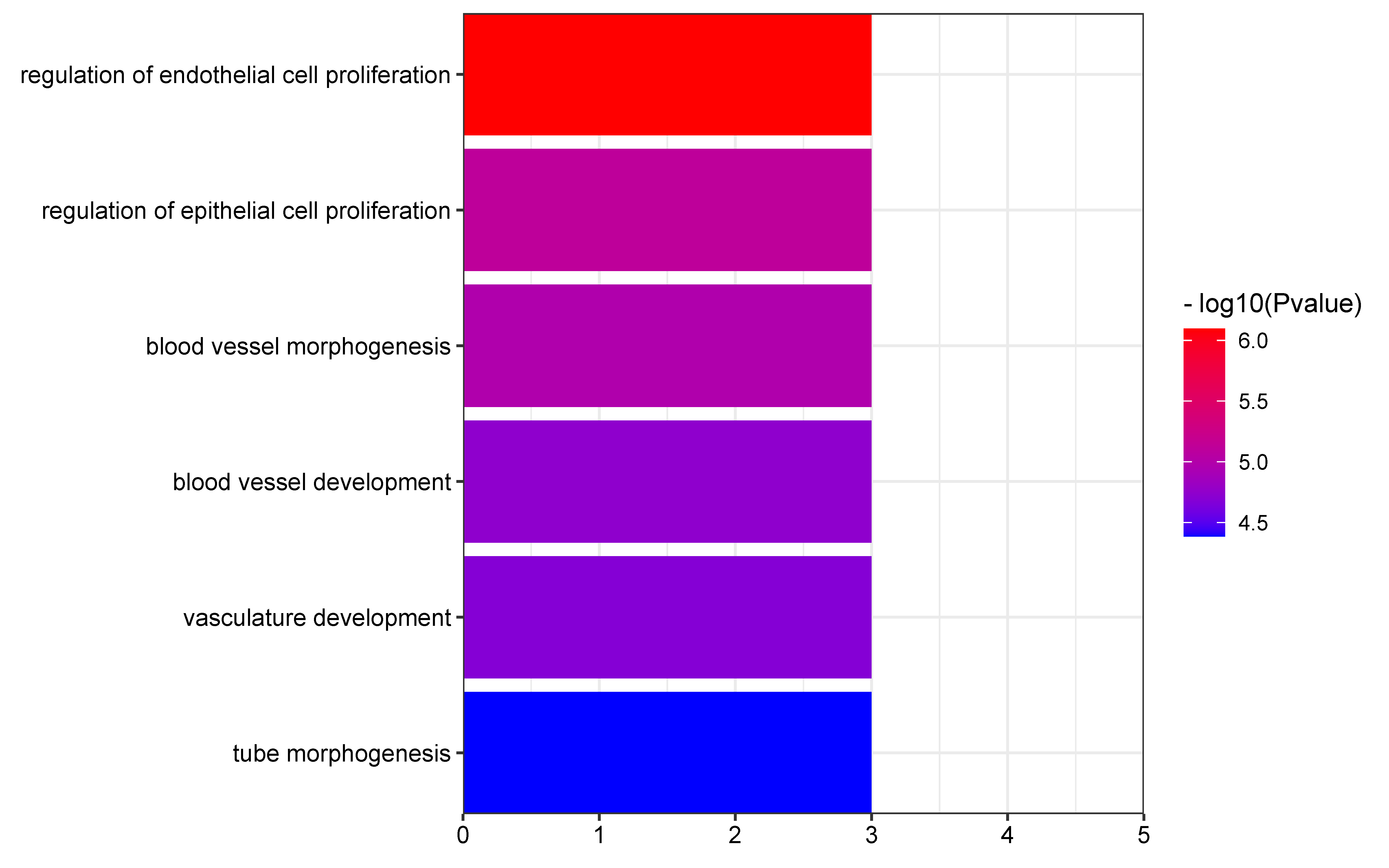

Supplement: Supplementary file 10 [file Image4.TIFF]
