# Supplementary material for: Identification of Angiogenesis-Related Prognostic Biomarkers Associated With Immune Cell Infiltration in Breast Cancer
Source: Front Cell Dev Biol. 2022 May 6;10:853324. doi: 10.3389/fcell.2022.853324 (PMC9121305; doi:10.3389/fcell.2022.853324)
Supplement: Supplementary file 12 [file DataSheet1.docx]

**Figure Legends for Supplementary Figures.**

**Figure 1S.** Development of risk score model based on the four ARGs signature in patients with breast cancer from GSE7390 datasets. (A) The risk score distribution and survival status of patients; (B) Kaplan–Meier analysis of the prognostic model; (C) Heatmap of the four ARGs expression and clinical parameter profiles between high- and low-risk groups; (D) Time-dependent ROC analysis of the risk score model.

**Figure 2S.** Development of risk score model based on the four ARGs signature in patients with breast cancer from GSE88770 datasets. (A) The risk score distribution and survival status of patients; (B) Kaplan–Meier analysis of the prognostic model; (C) Heatmap of the four ARGs expression and clinical parameter profiles between high- and low-risk groups; (D) Time-dependent ROC analysis of the risk score model.

**Figure 3S.** The relationship between ARGs-based risk score and clinical features including M stage (A), age (B), N stage (C), clinical stage (D), ER status (E) and PR status (F).

**Figure 4S.** Function enrichment of these four prognostic genes (TNFSF12, SCG2, COL4A3 and TNNI3).
